# Electrical impedance tomography imaging using *a priori *ultrasound data

**DOI:** 10.1186/1475-925X-5-8

**Published:** 2006-02-06

**Authors:** Manuchehr Soleimani

**Affiliations:** 1 William Lee Innovation Centre, School of Materials, The University of Manchester, Manchester M60 1QD, UK

## Abstract

**Background:**

Different imaging systems (e.g. electrical, magnetic, and ultrasound) rely on a wide variety of physical properties, and the datasets obtained from such systems provide only partial information about the unknown true state. One approach is to choose complementary imaging systems, and to combine the information to achieve a better representation.

**Methods:**

This paper discusses the combination of ultrasound and electrical impedance tomography (EIT) information. Ultrasound reflection signals are good at locating sharp acoustic density changes associated with the boundaries of objects. Some boundaries, however, may be indeterminable due to masking from intermediate boundaries or because they are outside the ultrasound beam. Conversely, the EIT data contains relatively low-quality information, but it includes the whole region enclosed by the electrodes.

**Results:**

Results are shown from a narrowband level-set method applied to 2D and 3D EIT incorporating limited angle ultrasound time of flight data.

**Conclusion:**

The EIT reconstruction is shown to be faster and more accurate using the additional edge information from both one and four transducer ultrasound systems.

## Background

Electrical impedance tomography (EIT) seeks to image electrical conductivity distribution of an object by measuring the impedance data between electrodes attached to the outer surface of the body [1]. In this paper we are developing an EIT imaging technique combined with a priori ultrasound data. Our proposed method is looking to a localised change in conductivity of part of the imaging area. The application of EIT envisaged in this paper, as motivation, is monitoring of cryosurgery. Cryosurgery is a minimally invasive way of destroying the undesired tissues by freezing them down to between -20 to -80 degree C [2,3]. Feasibility of EIT for Cryosurgery monitoring has been studied in [4,5]. In this application a probe is inserted into cancerous tissue. An ice ball forms around the probe destroying the surrounding tissue. It is very important to monitor the location, size and shape of the ice ball, especially if the area to be destroyed is delicate and contains critical tissue. This monitoring is usually achieved using magnetic resonance imaging (MRI) or CT fluoroscopy [6,7]. Both EIT [5] and ultrasound imaging [8] approaches offer cheaper systems, but individually do not provide sufficient detail to be of practical use.

A standard single transducer ultrasound system would typically only see one face of the ice ball. In contrast, a 3D EIT system would sense the whole volume but the boundaries of the ice ball would be difficult to accurately determine. Full medical ultrasound systems use scanning transducers or phased arrays to produce 2D or 3D images of the body. These systems are expensive and will still only show one face of the ice ball. A simpler and much cheaper system uses a few individual ultrasound transducers placed around the object. This method can estimate the edge position in a few places but cannot produce a full image. This information, however, could be used to improve the reconstruction method of an additional modality, such as EIT. This paper looks to introduce the fusion approach by proposing a narrowband level-set algorithm, which focuses the EIT reconstruction using the ultrasound data.

The level-set method was initially introduced for tracking propagating boundaries, but can also be used to locate static boundaries such as the interface between an inclusion and background [9-11]. There are alternative approaches for the shape reconstruction in EIT, including monotonicty method [11,12] geometrical based shape recovery [13-15]. Compare to geometrical modelling of the shape recovery [13-15], the level set method has an advantage that it handles multiple objects in an automatic fashion. Monotonicity method is fast and nonlinear, but provides only partial classification of the shape identification problem.

In this paper the finite element method (FEM) has been used to solve the forward problem of EIT. This is implemented in Matlab using codes from EIDORS2D and EIDORS3D [16,17]. A triangular mesh was used for the FEM model of the forward problem in 2D and a tetrahedral mesh was used in 3D. Using level set method allows us to use a dense mesh for the inverse problem (by applying a narrowband level set the size of the inverse to be solved remains small), consequently large scale forward problem needs to be solved. In order to improve the computational time of the forward solvers especially in 3D, we modified the EIDORS3D by applying algebraic multigrid method (AMG) [18] as a preconditioner for the conjugate gradient as the linear solver from the FEM model. Using AMG scheme improved the speed of the forward problem dramatically; so many forward problems could be solved as needed in level set method. In our modified version of EIDROS3D we developed a nonlinear EIT reconstruction and also minimized the computational time for calculating the Jacobian matrix, so it can be update many times.

Using an iterative method, with an update formula for the level-set function, the interface between two materials can be recovered. The level set is known to generate good interface results [9,10] but the computational cost is high, as it will need many iterations (hundreds of nonlinear steps). The number of iterations is a function of the initial guess for the shape as well as the complexity of the objects to be reconstructed. In this article we are using a priori ultrasound data to improve our initial guess for the object location, by identifying some points at the boundary of the object.

## Method

In EIT, the differences in the electrical properties, i.e. conductivity distribution inside the object; is used to generate a tomographic image. EIT is used in both medical and industrial applications. The advantage of such a technique over other imaging modalities is such that, it provides a non-invasive ("non-destructive" in an industrial terminology) method and requires no ionizing radiation. Furthermore, EIT is a relative low cost and simple functional technique. Moreover, a portable measurement system could also be designed for it. The most important drawback of EIT is its poor image resolution, which is often restricted by the number of electrodes used for data acquisition. Data acquisition is typically made by applying an electrical current to the object using a set of electrodes, and measuring the developed voltage between other electrodes.

EIT is suggested to be a good technique for the cryosurgery monitoring. In this article we are reformulating the EIT image reconstruction problem to an interface reconstruction problem between frozen and normal tissue. That would allow us to extract the information we need in more efficient way. Fortunately, this is an application of EIT that the conductivity contrast of the region to be imaged is high and a localised problem as the cryogen probe can tell us where the approximate location of the frozen area is. Here we are attempting to use some additional information about the interface by using ultrasound data.

### Ultrasound transducers

The ultrasound method is a single pulse-echo measurement [19]. The travel time from excitation of the transducer to receiving the reflected signal is recorded. The velocity of the ultrasound wave within the fluid is dependent on its compressibility and density. The distance travelled is calculated from the *time of flight (TOF) *of the ultrasound wave from transducer to the object and back again, and the known velocity of sound in the liquid. Ultrasound technique is based on measuring the TOF of ultrasonic waves arriving at the boundary of the region of interest, which here is the ice ball. Ultrasonic transducers are evenly spaced around the circumference of a body.

**Figure 1:**
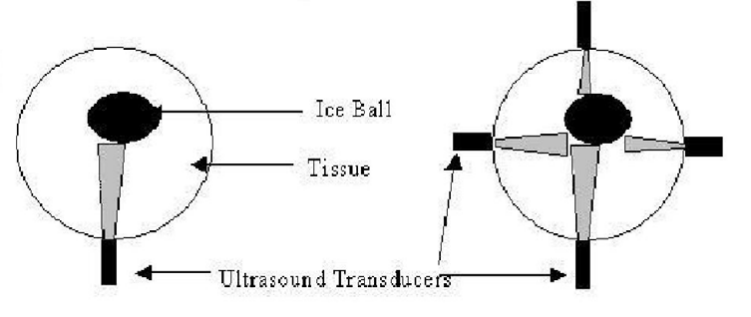
Diagram of ultrasound probing using a single transducer (left) and multiple transducers (right).

We assume the transducer beam is not diverging (near field, here we assume the probes are at the surface of the body) such that only a known narrow strip in front of the transducer is investigated (see Figure 1). The ultrasound signal therefore provides the position of the ice front within this narrow strip. This information is used in the electrical resistance reconstruction. More transducers could be incorporated to estimate the whole ice boundary from at other positions.

In normal medical ultrasound the tissue temperature is approximately constant throughout the body and the velocity is close to that of pure water. However in the cryogenic freezing application there will be noticeable temperature gradient. Since the velocity dependence of water is approximately 3 m/s per degree C, this variation in temperature will have a noticeable effect. It would be possible to model this temperature gradient and variation to calculate the velocity profile and then estimate the ice ball position. However to model the temperature distribution the ice ball position must be known, which leads to an iterative calculation. Another method would be to perform ultrasound measurements across the tissue from known positions on the skin surface. This would aid in the estimation of the velocity and temperature gradients.

### Level set method

The level-set technique is chosen to describe changing shapes since this method is able to easily model topological changes of the boundaries. In the shape reconstruction approach, it is assumed that the approximate values of background parameters and parameters inside the inclusions are known, but that the number, topology and shapes of the inclusions are unknown and have to be recovered from the data. Compared to the more typical pixel/voxel-based reconstruction schemes, the shape reconstruction approach has the advantage that the prior information about the high contrast of the inclusions is incorporated explicitly in the modelling of the problem. In a pixel/ voxel-based reconstruction scheme the approximate locations of the unknown inclusions are found during the early iterations, but it typically takes a large number of additional iterations to achieve accurate information concerning the precise shapes of these objects.

**Figure 2:**
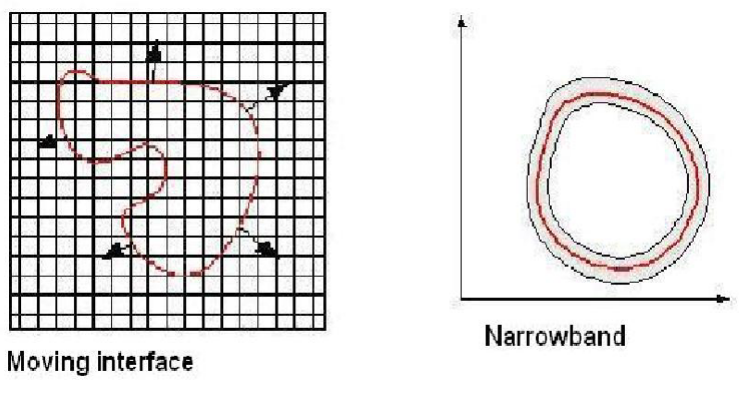
Diagram showing the moving boundary (left) and narrowband (right) around the interface in the level-set approach.



Figure 2 schematically shows a moving boundary and a narrowband at the interface. Here, the equation describing the moving fronts is

where *F *is the speed function and Φ is the boundary at time t.

We do not use equation (1) to describe the front propagation. Instead we have implemented a narrowband level set method [10]. In narrowband level set method we use an iterative optimisation technique and in each iteration the inverse problem has been solved on the interface between two-phase materials [10].

The conductivity at each point *r *can be described in terms of the level-set function depending on the position of the point *r *with respect to the boundary ∂*D *of the inclusion D as



and

∂*D *= {*r*:Φ(*r*) = 0}.     (3)

Here σ_int _and σ_*ext *_are the conductivity of the inclusion and the background respectively. The describing level-set function is a function form *R*^3 ^→ *R *for three-dimensional case, and its value is zero on the boundary, it has a negative sign inside and a positive sign outside of the boundary.

The inverse boundary value problem is to find the boundary ∂*D *(which in turn describes a conductivity distribution) that minimizes the mismatch between the measured and fitted voltage data. The mismatch function, Ψ(∂D), is defined as

Ψ(∂*D*) = ||*V*_*m *_- *V*(∂*D*(σ))|| + *G*(∂*D*(σ))     (4)

where *V*_*m *_are the measured voltages, and *V*(∂*D*(σ)) are the voltages calculated from the conductivity distribution, σ, derived from the corresponding boundary ∂*D *and *G*. is a regularisation term applied to the interface. In this paper the regularisation matrix is the identity matrix. The derivative of the voltage to a change in interface was derive [9] and [10,11] and has been used in this study. Regularised Gauss-Newton update formula [11] has been used to reconstruct the interface. Small values of the mismatch between measured and simulated voltage indicates good locations for the boundary where as large values indicate poor estimation of the boundary. In particular, the boundary which makes this as small as possible is used, along with the inclusion and background resistivities, to give the level-set reconstruction. In practice this minimum cannot be found in a closed form and so a numerical procedure is required. To perform the minimization the following algorithm [10] is used

1. Start with an initial guess for the shape of the inclusion, which is an initial level-set function, in our case a circle located in the centre.

2. Define the interface and narrow band; the narrow band is an area that includes pixels sharing points with the interface.

3. Solve the forward problem and calculate the Jacobian with respect to the boundary.

4. Update the level-set function and calculate a new interface boundary and narrow band.

5. Check the misfit in the data and if the error is small enough then stop.

6. If the misfit is not small go to Step 2

## Results

**Figure 3:**
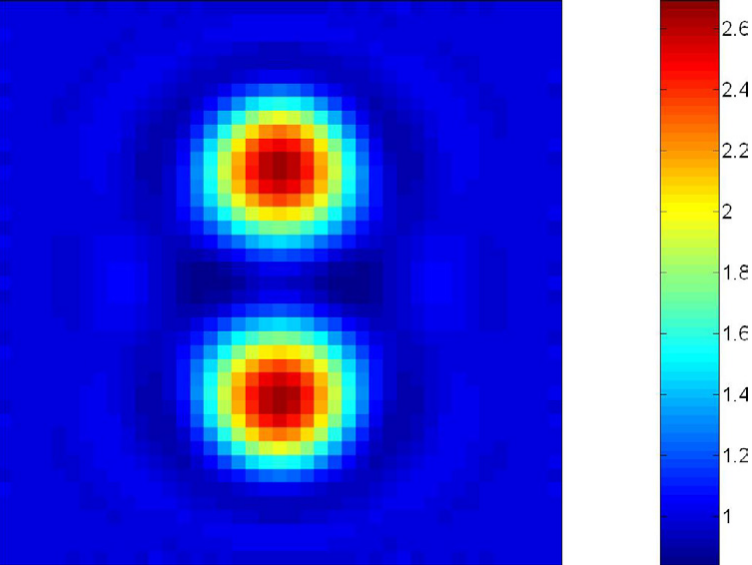
The reconstruction of a high resolution EIT image suitable for cryosurgery monitoring using simulated data.

In general for EIT to be used in cryosurgery monitoring a higher resolution EIT image is needed. Figure 3 shows the simulation of EIT reconstruction for cryosurgery using 64 electrodes (this image has been provided by John Edd [20] from university of California Brekely). It is a reconstruction of the change in conductivity upon thawing of a dual cryoprobe cryosurgical treatment. There are two circular regions of ablated tissue (each having a conductivity approximately 2.43 times that of normal tissue), while the surrounding tissue is healthy (step transition). The image contains 41 × 41 square pixels and uses 64 electrodes (1952 measurements). To improve the EIT image resolution one can increase number of electrodes but there will be limited number of electrodes depending on the accuracy of the measurement system. As it has been shown in our proposed method, the narrowband level set representation of the inverse EIT problem can improve the accuracy of the interface recovery.

**Figure 4:**
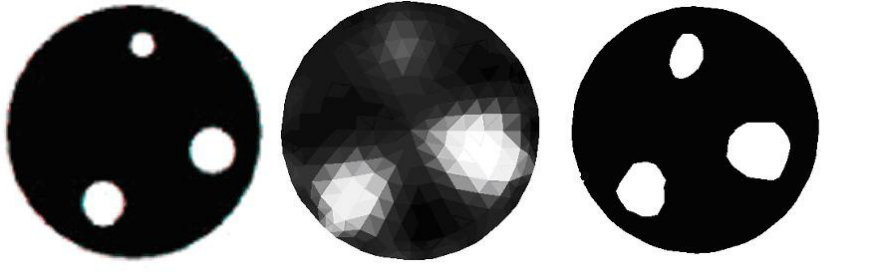
A 2D example with the truth with three inclusions (left), a standard reconstruction (centre) and a level-set reconstruction (right).

Figure 4 shows a comparison between pixel-based image reconstruction [16,17] and the level-set method. On the left hand side is true conductivity distribution used to represent the measurement. The figure at the centre shows the pixel-based image reconstruction and on the right is the level-set reconstruction. It is clear from this experimental test example that the level-set method outperforms the standard image based method. Cylindrical 3D objects were used for gathering the data experimentally, and data was collected from the experimental EIT (16 electrodes EIT) system was designed and built at the Univesity of Kaipio in Finland [16].

**Figure 5:**
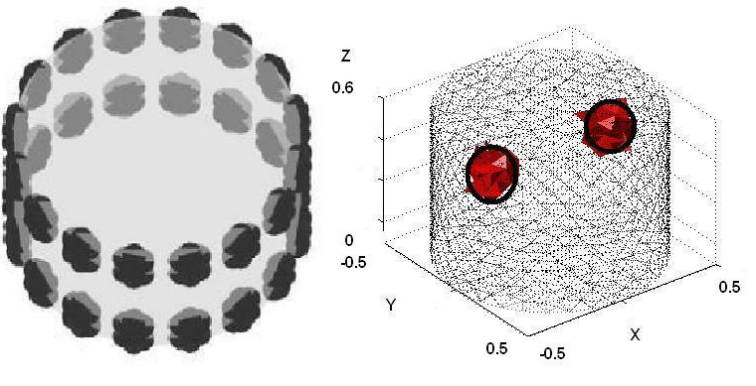
A 3D example with two-plane electrode arrangement (left), truth with two inclusions (centre) and level-set reconstruction (right).

Figure 5 shows an example for 3D reconstruction with a simulated 3D tank. There are 16 electrodes in two rings of electrodes. This model has been chosen to demonstrate the simulation study. In any particular application one needs to tack into account the geometry of the object accordingly. The inverse mesh includes 9423 elements and solving the full inverse problem of the pixel-based method is very time consuming. Using the level-set method we have solved several smaller sized inverse problems resulting in an overall faster algorithm. Size of the inverse problem to be solved in iterations of the narrowband level set method was an average number of 1023. Reduction of the size of the inverse problem reduces the memory required to solve the inverse problem and also computational time required to solve the inverse problem. The number of iterations in level set method is normally higher compared to the number of iterations used in traditional pixeled based image reconstruction [11]. There can be two reasons for that. First if the inclusions are far from the initial guess it may take iterations to find the location of an inclusion by an interface search method. Secondly if the shape of the inclusions is complicated, it will take much iteration to reconstruct the shape of the object. In this study we don't have a solution to reduce the number of level set iterations if the shape is complicated. By using a priori ultrasound knowledge about the boundary of the object we can speed up the level set algorithm to converge in less iteration.

**Figure 6:**
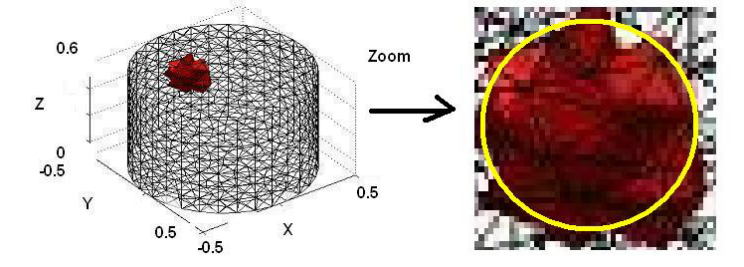
True position of the inclusion and reconstruction using level set method.

For enhanced EIT reconstruction using single ultrasound transducer measurements we assume that the bottom edge position of the ice ball is known. For the multiple transducer-enhanced reconstructions we include four edge positions as shown on the right in Figure 6.

**Figure 7:**
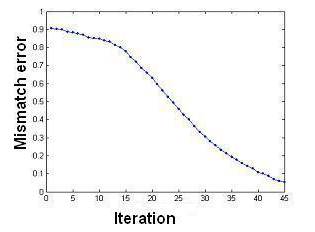
The reduction in the mismatch error for non-enhanced reconstruction.

The number of iterations in the level-set method is a function of the initial guess. If the initial guess is far from the real object it will take more iteration to recover the shape. The real object (see Figure 7) is a spherical object centred at (0.4, 0, 0.4) m with the diameter of 0.1 m.

**Figure 8:**
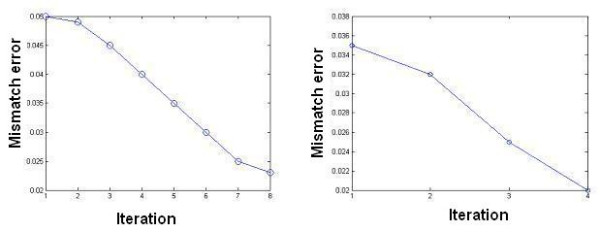
The reduction in the mismatch error employing ultrasound edge information from one transducer (left) and four transducers (right).

To assess and compare the procedures the mismatch error, Ψ, is calculated at each iteration. The convergence plot in Figure 8 shows that, for a level-set reconstruction when the initial guess was a sphere with diameter 0.07 m centred at (0.1, 0, 0.1) m, the number of iterations was high. This is because during the evolution of the level-set function the object moves within a low sensitivity region – that is between the two planes of electrodes in the horizontal direction.

Using a single ultrasound transducer a point, (0.3, 0, 0.4) m, on the boundary of the inclusion is identified and used to help start the level-set algorithm. The initial guess is of a spherical object with diameter 0.07 m centred at (0.3, 0, 0.33) m. The convergence improves (see Figure 8 left) and in a few iterations a very good estimate of the true object can be reconstructed.

Further improvement, both in terms of the speed of the convergence and the accuracy of the reconstructed shapes, is expected with multiple ultrasound transducers. To investigate this, ultrasound probes help to provide the positions of four points in object boundary. A spherical object passing through these four points is implemented as the initial guess. The convergence of the level-set method is faster as expected (Figure 8 right), however the shape reconstruction accuracy does not improve significantly over the single transducer results. This is likely to be due to the coarseness of the level-set mesh. The smallest size of the ice ball that can be reconstructed depends on the accuracy of the measurement system and computational model. In [18] for brain cryosurgery, we have shown that the voltage differences between frozen and normal tissues in mv are proportional to the volume of ice ball in cm^3^. On the other hand the choice of mesh density and size of narrowband has an impact in accuracy of the interface.

## Conclusion

Shape identification is an inverse boundary value problem; therefore it is not efficient to use the common image reconstruction methods. Shape reconstruction in 3D EIT is presented in this paper. The main advantage of the level-set formulation is that at each iteration the inverse problem needs to be solved in the interface between two materials rather than in the whole region of interest. In terms of including prior information, the level-set method incorporates important regularization, namely knowledge of the two-phase material. Additional information was included from the ultrasound data, and an improvement in the speed of convergence and accuracy of the results by this data fusion has been discussed. Finally, the 3D EIT level-set inversion can be improved both in speed and accuracy by incorporating ultrasound time of flight data. It worth noticing that the technique developed in this paper is a localised EIT reconstruction, concentrating in a small region, any change or uncertainty on conductivity of the rest of the imaging area will have an effect on the estimation of the interface, we acknowledge that. This is still an open debate in EIT community how trustable is localised information gathered by an EIT system without a whole EIT image. The question to be answered is in EIT we have limited resources in terms of measured data, if we want to include many information to be identified by EIT data, we can calculate them lesser accurate, but if we assume some of the information, we might be simply wrong in some of those assumptions. In our continued study we will further investigate the validity of the localised information especially when a second modality is also involved. We suggest following steps as the best strategy to be used in a practical application

1. Generate a pixel based EIT image (using a linear image reconstruction).

2. Evaluate an approximated speed profile for the ultrasound based on conductivity profile (approximate it to temperature gradient) generated using linear EIT image and consider the speed with different organs based on EIT image.

3. Calculate some points at the boundary of the inclusion (here is frozen tissue) using ultrasound data.

4. Applying narrowband level set method as a more accurate interface reconstruction technique using EIT data and a priori ultrasound data.

With above strategy, we have an EIT image but with less accurate interface information and a localised conductivity interface reconstruction with more detailed information about the interface between frozen and normal tissues. Combination of full ultrasound tomography with consideration of different speed of ultrasound in different organs and different temperature is our main aim for future study.
